# Computation and measurement of cell decision making errors using single cell data

**DOI:** 10.1371/journal.pcbi.1005436

**Published:** 2017-04-05

**Authors:** Iman Habibi, Raymond Cheong, Tomasz Lipniacki, Andre Levchenko, Effat S. Emamian, Ali Abdi

**Affiliations:** 1 Center for Wireless Information Processing, Department of Electrical and Computer Engineering, New Jersey Institute of Technology, Newark, NJ, United States of America; 2 Department of Biomedical Engineering, Johns Hopkins University, Baltimore, MD, United States of America; 3 Institute of Fundamental Technological Research, Polish Academy of Sciences, Pawinskiego 5B, Warsaw, Poland; 4 Yale Systems Biology Institute and Department of Biomedical Engineering, Yale University, New Haven, CT, United States of America; 5 Advanced Technologies for Novel Therapeutics, Enterprise Development Center, New Jersey Institute of Technology, Newark, NJ, United States of America; 6 Department of Biological Sciences, New Jersey Institute of Technology, Newark, NJ, United States of America; Princeton University, UNITED STATES

## Abstract

In this study a new computational method is developed to quantify decision making errors in cells, caused by noise and signaling failures. Analysis of tumor necrosis factor (TNF) signaling pathway which regulates the transcription factor Nuclear Factor κB (NF-κB) using this method identifies two types of incorrect cell decisions called *false alarm* and *miss*. These two events represent, respectively, declaring a signal which is not present and missing a signal that does exist. Using single cell experimental data and the developed method, we compute false alarm and miss error probabilities in wild-type cells and provide a formulation which shows how these metrics depend on the signal transduction noise level. We also show that in the presence of abnormalities in a cell, decision making processes can be significantly affected, compared to a wild-type cell, and the method is able to model and measure such effects. In the TNF—NF-κB pathway, the method computes and reveals changes in false alarm and miss probabilities in A20-deficient cells, caused by cell’s inability to inhibit TNF-induced NF-κB response. In biological terms, a higher false alarm metric in this abnormal TNF signaling system indicates perceiving more cytokine signals which in fact do not exist at the system input, whereas a higher miss metric indicates that it is highly likely to miss signals that actually exist. Overall, this study demonstrates the ability of the developed method for modeling cell decision making errors under normal and abnormal conditions, and in the presence of transduction noise uncertainty. Compared to the previously reported pathway capacity metric, our results suggest that the introduced decision error metrics characterize signaling failures more accurately. This is mainly because while capacity is a useful metric to study information transmission in signaling pathways, it does not capture the overlap between TNF-induced noisy response curves.

## Author summary

Cell continuously receives signals from the surrounding environment and is supposed to make correct decisions, i.e., respond properly to various signals and initiate certain cellular functions. Modeling and quantification of decision making processes in a cell have emerged as important areas of research in recent years. Due to signal transduction noise, cells respond differently to similar inputs, which may result in incorrect cell decisions. Here we develop a novel method for characterization of decision making processes in cells, using statistical signal processing and decision theory concepts. To demonstrate the utility of the method, we apply it to an important signaling pathway that regulates molecules which play key roles in cell survival. Our method reveals that cells can make two types of incorrect decisions, namely, false alarm and miss events. We measure the likelihood of these decisions using single cell experimental data, and demonstrate how these incorrect decisions are related to the signal transduction noise or absence of certain molecular functions. Using our method, decision making errors in other molecular systems can be modeled. Such models are useful for understanding and developing treatments for pathological processes such as inflammation, various cancers and autoimmune diseases.

## Introduction

Each individual cell receives signals from the surrounding environment and is supposed to respond properly through a variety of biochemical interactions among its signaling molecules. Single cell studies and modeling approaches have emerged in recent years [1,2,3], to understand the biochemical processes in each individual cell, as opposed to a large population of cells and their average behavior. Due to signal transduction noise, a cell can respond differently to the same input, which may result in incorrect (unexpected) cell decisions and responses [2]. Upon providing an input signal, however, it is not clear whether the cell is going to make a correct decision or not. Due to the random nature of the transduction noise, this decision making becomes somewhat probabilistic [2]. Here we introduce a method for characterization and quantification of decision making processes in cells, using statistical signal processing and decision theory concepts [4] used in radar and sonar systems. The basic goal of such systems is the ability to correctly decide on the presence or absence of an object. For example, in a radar system it is of interest to decide if there is an object transmitting a constant signal, while noise is present. If the received signal is much stronger than noise, the system can correctly declare the presence of the object. However, if the received signal is much weaker than noise, the system will miss the presence of the object. This erroneous decision is called a *miss* event. The radar system can make another type of erroneous decision, called a *false alarm* event, where there is no object but noise misleads the system to falsely declare the presence of an object. A mathematical model for this example [4], including received signal and noise models, the decision making algorithm, probabilities for making incorrect decisions and some numerical results are presented in Materials and Methods.

**Fig 1:**
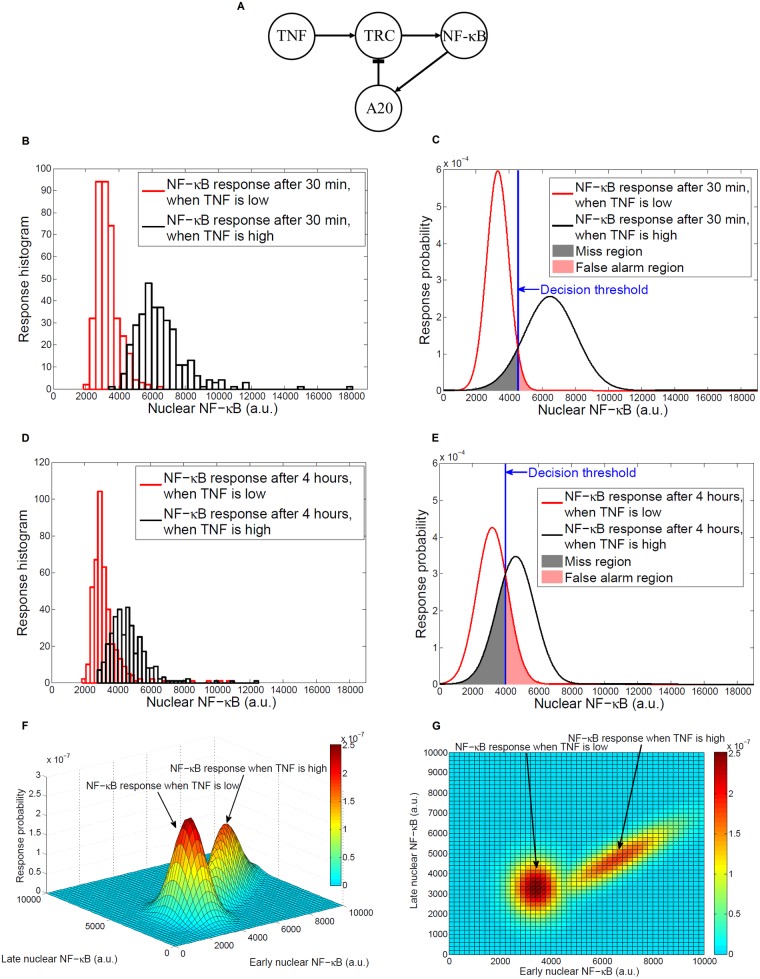
Cell decision making processes in the TNF—NF-κB signaling system. (**A**) The pathway. (**B**) Histograms of NF-κB responses of hundreds of cells to low and high TNF levels after 30 minutes. (**C**) Gaussian probability density functions for NF-κB responses to low and high TNF levels after 30 minutes. The blue vertical line represents the maximum likelihood decision threshold that minimizes *P*_*e*_, the overall probability of error in making decisions. Pink and gray regions around the decision threshold represent false alarm and miss decisions. (**D**) Histograms of NF-κB responses of hundreds of cells after 4 hours. (**E**) NF-κB response curves, maximum likelihood decision threshold, false alarm and miss decision regions after 4 hours. (**F**) Bivariate Gaussian curves for NF-κB responses at the two time points 30 minutes (early) and 4 hours (late). (**G**) Top view of bivariate Gaussian response curves for NF-κB.

To explain the method in a practical way and in the context of molecular computational biology, we use the tumor necrosis factor (TNF) signaling pathway [2] which regulates the transcription factor nuclear factor κB (NF-κB) (**Fig 1A**). NF-κB is a nuclear transcription factor that regulates numerous genes which play important roles in cell survival, apoptosis, viral replication, and is involved in pathological processes such as inflammation, various cancers and autoimmune diseases. In the TNF signaling pathway (**Fig 1A**), the molecule A20 has an inhibitory feedback effect, whereas TRC stands for the TNF receptor complex [2]. TNF is a cytokine that can mediate both pro-apoptotic and anti-apoptotic signals [5]. In wild-type cells and upon binding of TNF ligands, NF-κB translocates to the nucleus, temporarily increasing the level of nuclear NF-κB. NF-κB activation rescues the cell from apoptosis. Then due to the negative feedback of A20, the nuclear NF-κB level decreases. This short period of NF-κB activity is sufficient to activate transcription of the so called early genes, including numerous cytokines and its inhibitor A20. In A20-deficient cells, the level of nuclear NF-κB remains relatively high for several hours. Loss or mutation of A20 can result in chronic inflammation and can promote cancer [6,7].

The signal transduction noise considered in our analysis encompasses all factors that make cell responses to the same signal variable or heterogeneous. In reference [3] it is demonstrated that both intrinsic and extrinsic noise contribute to the transduction noise in the NF-κB pathway. Extrinsic noise results from the fact that at the time of stimulation, cells are not identical and may have different levels of TNF receptors and other components of the signal transduction cascade. Intrinsic noise, on the other hand, results from the randomness of the biochemical reactions that involve a small number of molecules.

## Results and discussion

Recent information theoretical analysis of single cell data has demonstrated that in the TNF signaling pathway, cell can only decide whether TNF level at the system input is high or low [2]. In other words, based on the nuclear NF-κB level, cell can only tell if there is high TNF level at the input or not [2]. During this process, we formulate that cell can make two types of incorrect decisions: deciding that TNF is high at the system input whereas in fact it is low, or missing TNF’s high level when it is actually high. These two incorrect decisions can be called false alarm and miss events, respectively, similarly to the terminology used in radar and sonar [4]. The likelihood of occurrence of these incorrect decisions depends on the signal transduction noise. To understand how cell makes a decision on whether TNF is high or low, we first studied two TNF concentrations of 8 and 0.0021 ng/mL, respectively (other TNF levels are discussed later). The histograms representing NF-κB responses of hundreds of cells to each TNF stimulus after 30 minutes are shown in **Fig 1B**. By using a probability distribution such as Gaussian (**Fig 1C**) (see Materials and Methods) for histograms, we specified the regions associated with incorrect decisions (**Fig 1C**) (see Materials and Methods). These regions are determined by the optimal decision threshold obtained using the maximum likelihood principle^‎4^ (see Materials and Methods), which simply indicates that the best decision on some possible scenarios is selecting the one that has the highest likelihood of occurring [4]. The area to the right of the decision threshold under the low TNF response curve is the *false alarm* region (**Fig 1C**), meaning that nuclear NF-κB level could be greater than the threshold due to the noise, which falsely indicates a high level of TNF at the system input. The size of this shaded area specifies *P*_*FA*_, the false alarm probability. On the other hand, the area to the left of the decision threshold under the high TNF response curve is the *miss* region (**Fig 1C**), meaning that due to the noise, nuclear NF-κB level could be smaller than the threshold, which results in missing the presence of high TNF level at the system input. The size of this shaded area is *P*_*M*_, the miss probability. Using the single cell experimental data we calculated *P*_*FA*_ = 0.04 and *P*_*M*_ = 0.1 (see Materials and Methods). The higher value for *P*_*M*_ can be attributed to the broader response curve when TNF is high (**Fig 1C**). The overall probability of error *P*_*e*_ for making a decision is given by *P*_*e*_ = (*P*_*FA*_ + *P*_*M*_)/2 = 0.07 (see Materials and Methods), which is the average of false alarm and miss probabilities.

We also collected the histograms of NF-κB responses of hundreds of cells to each TNF stimulus after 4 hours (**Fig 1D**), which seem to have more overlap, compared to the response histograms collected at 30 min. This can be better understood by looking at the two response curves and the larger false alarm and miss regions (**Fig 1E**). In fact, we observed higher values for false alarm and miss probabilities, i.e., *P*_*FA*_ = 0.2 and *P*_*M*_ = 0.29 (see Materials and Methods). These higher values for false alarm and miss probabilities, as well as the higher overall probability of error *P*_*e*_ = (0.2 + 0.29)/2 = 0.245 can be due to the negative feedback of A20 (**Fig 1A**), which reduced the level of nuclear NF-κB in 4 hours, when TNF was high (notice the considerable shift of the TNF-high response curve to the left that we observe in **Fig 1E**, compared to **Fig 1C**). To understand the decision making process based on both early and late responses, we computed (see Materials and Methods) high and low TNF joint response curves of the nuclear NF-κB at 30 minutes and 4 hours (**Fig 1F**). The top view of the response curves (**Fig 1G**) shows that while high and low TNF concentrations produce relatively distinct distribution patterns in the early response domain, they have a higher degree of overlap in the late response domain. Using a more sophisticated approach to determine decision thresholds and decision probabilities based on joint early and late response data (see Materials and Methods), we calculated *P*_*FA*_ = 0.03, *P*_*M*_ = 0.1 and *P*_*e*_ = 0.065. These results turned out to be about the same as early decision probabilities, i.e., *P*_*FA*_ = 0.04, *P*_*M*_ = 0.1 and *P*_*e*_ = 0.07. It appears that in this signaling pathway, maximum likelihood decisions based on joint early/late events and early event alone provide the same finding on whether TNF level at the system input is high or low.

**Fig 2:**
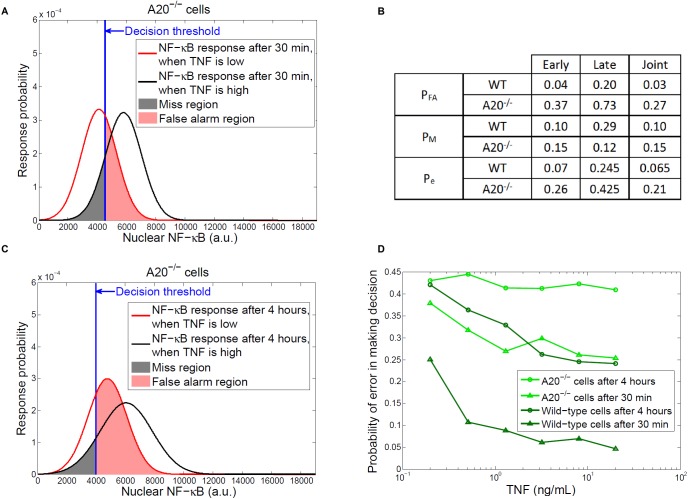
Decisions in A20-deficient cells. (**A**) Gaussian probability density functions for NF-κB responses to low and high TNF levels after 30 minutes in A20^-/-^ cells. The blue vertical line represents the decision threshold of the wild-type case after 30 minutes, considering that A20-deficient cells are unaware of the deficiency and therefore erroneously utilize the previously used threshold (The developed method is not limited to this choice and certainly other thresholds can be used. To reflect the fatality caused by A20 deficiency [8], in our model A20^-/-^ cells make decisions using an incorrect decision threshold, i.e., the threshold that was used before the occurrence of the deficiency). Pink and gray regions around the decision threshold represent false alarm and miss decisions. The density functions are obtained from histograms of NF-κB responses of hundreds of cells to low and high TNF levels after 30 minutes in A20^-/-^ cells. (**B**) False alarm, miss and overall decision error probabilities in wild-type (WT) and A20^-/-^ cells. (**C**) NF-κB response curves, decision threshold, false alarm and miss decision regions after 4 hours in A20^-/-^ cells. The response curves are obtained from histograms of NF-κB responses of hundreds of cells after 4 hours in A20^-/-^ cells. (**D**) Overall probability of error in making decisions to distinguish between 0.0021 ng/mL dose and higher doses, as a function of the higher dose, after 30 minutes or 4 hours of TNF stimulation, in wild-type and A20^-/-^ cells.

In the presence of abnormalities in a cell, such decision making processes can significantly change, compared to a wild-type cell. For example, in the absence of A20, a cell is unable to inhibit the TNF-induced NF-κB response [2,8]. Under this condition, response curves of hundreds of A20^-/-^ cells to high and low TNF levels after 30 minutes (**Fig 2A**) show significant overlap, compared to the response of wild-type cells (**Fig 1C**). This is because the negative feedback was no longer present in A20^-/-^ cells, which resulted in the broadening of the TNF-low response curve and the increase in its mean value (**Fig 2A**). Therefore, the false alarm and miss regions in A20^-/-^ cells turned out to be much larger (**Fig 2A**), for which we computed *P*_*FA*_ = 0.37 and *P*_*M*_ = 0.15 (see Materials and Methods). Both false alarm and miss probabilities were greater than those of wild-type cells (**Fig 2B**). In biological terms, the higher false alarm rate in this abnormal TNF signaling system means perceiving more signals which in fact do not exist at the system input, whereas the higher miss rate indicates that it is more likely to miss signals that actually exist.

Using the response curves after 4 hours in A20^-/-^ cells (**Fig 2C**), we computed *P*_*FA*_ = 0.73 and *P*_*M*_ = 0.12 (see Materials and Methods). The increase in *P*_*FA*_ and decrease in *P*_*M*_, compared to the wild-type cells, reflected a more profound effect of the lack of negative feedback after 4 hours in A20^-/-^ cells, which resulted in an increase in the mean nuclear NF-κB level for both low and high TNFs (**Fig 2C**). Computations using both early and late response data (see Materials and Methods) revealed that in this signaling pathway, decisions based on joint early/late events and early events in A20^-/-^ cells provide about the same results and probabilities on whether TNF level at the system input is high or low (**Fig 2B**).

To study the impact of different TNF concentrations on cell decisions, we computed the overall probability of error *P*_*e*_ in making decisions after 30 minutes and 4 hours in both wild-type and A20^-/-^ cells (**Fig 2D**), after treatment with six different TNF concentrations. This analysis shows that in wild-type cells a higher decision error rate *P*_*e*_ is observed over time for all TNF concentrations. Also in wild-type cells *P*_*e*_ decreases as TNF concentration increases up to about 3 ng/mL, and then becomes less sensitive to the higher concentrations of TNF.

On the other hand, depletion of A20 increases the decision error rate *P*_*e*_, compared to the wild-type cells, after 30 minute treatment (**Fig 2D**). Interestingly, A20^-/-^ cells show higher *P*_*e*_ after the 4 hour treatment that is nearly insensitive to the increase in TNF concentration. Overall, for each time course, there is a significant increase in *P*_*e*_ in A20^-/-^ cells, compared to wild-type cells (**Fig 2D**). This is because of the failure of the signaling pathway due to A20 deficiency, where cells fail to stop TNF-induced NF-κB response. This observation further confirms the usefulness of the decision error rate *P*_*e*_ as a metric and method for modeling and measuring cell decision making processes under normal and abnormal conditions and in the presence of transduction noise uncertainty.

## Extensions to more complex settings and broader signaling contexts

The developed approach can be extended to more complex and larger signaling networks, where inputs could be ligands or secondary messengers, and outputs could be several transcription factors that produce certain cellular functions [9]. Then by analyzing the concentration levels of these transcription factors at single or multiple time points using the proposed approach, probabilities of various cell fates in response to the input signals can be computed.

In a broader context, one notes that in various organisms ranging from simple ones such as viruses to bacteria, yeast, lower metazoans and finally complex organisms such as mammals, various decisions are made in the presence of noise [10]. Depending on the concentration levels of certain molecules and their changes, regulated by some intracellular molecular networks, a cell may select from several possible fates. For example, in embryonic stem cells in mammals, the Nanog transcription factor expression level, which might be affected by molecular noise, is a determinant of cell differentiation, if proper signals are present [10]. In this context, one can use the approach presented here to compute false alarm and miss probabilities at different time instants, to better understand how precise or erroneous the decision to differentiate is (given that noise is present), and how it changes over time. In a broader context, one may envision studying cell decision making processes in other organisms, such as those reviewed in [10], using the developed approach.

## Comparison with other approaches

### Capacity

This study shows that compared to the overall probability of error *P*_*e*_ introduced in this paper for signaling systems, the signaling capacity defined as the maximum amount of information between the system input and output, may not be a convenient metric for revealing dysfunctionalities in the system. The rationale is that while in the TNF—NF-κB pathway (**Fig 1A**) a reduction in capacity is observed in A20^-/-^ cells in 30 minutes, compared to wild-type cells, an opposite effect, i.e., capacity increase, is observed after 4 hours [2]. Therefore, the impact of A20 deficiency on the pathway capacity appears in different directions over time. The introduced error probability metric, on the other hand, consistently shows the increased level of erroneous behavior of this signaling pathway, in both short and long terms.

The difference between decision error probability and capacity in the context of dysfunctionalities can be anticipated. This is because decision error probability is a metric defined such that it directly reflects departure of the pathway from normal behavior and its expected response. Capacity, on the other hand, is defined to measure the maximum amount of information that can flow from the pathway input to its output. While, in general, one may expect that a higher capacity in a pathway is a desired outcome, one can also note that the increased capacity might be caused by an alteration or loss of some otherwise important molecular functions in the pathway. In the TNF—NF-κB pathway, it has indeed been observed [2] that after 4 hours, A20-deficient cells exhibit a higher capacity, compared to wild-type cells. The point we are making here is that the higher amount of information that can travel from TNF to NF-κB in A20-deficient cells may not necessarily reflect biologically appropriate functioning of the pathway. To be able to understand dysfunctionalities in a pathway and how they affect cell decision makings, one can therefore benefit from a complementary metric and approach to characterize cell decision making errors in abnormal pathways, which we have studied here.

In summary, capacity is a useful metric for studying information transmission in signaling pathways, whereas the introduced metrics of false alarm, miss and overall error rates are suitable for modeling decision making errors caused by noise and signaling failures.

### Dynamical modeling

The goal of dynamical modeling is to use tools such as differential equations or stochastic processes, to model changes in the concentration levels of molecules with time. On the other hand, our approach aims at statistical characterization of decision making processes in cells, based on the concentration levels of certain molecules that control cell decisions, using statistical signal processing and decision theory tools. The concentration levels can be obtained via either experiments or stochastic simulations. As an example, in reference [3] a stochastic dynamical model is developed, which mimics nuclear NF-κB level changes with time, in response to a given TNF dose. The model is designed to assess the kinetics of molecular activities in a representative cell, provides information about single cell responses, and can also be used to simulate distributions of given protein levels across a population. It does not quantify the chance of missing a signal. The proposed approach provides methods to analyze single cell data in the context of cell decision making. For example, TNF high level of 8 ng/mL indicates the presence of a strong signal. However, due to noise, there is a chance for a cell to miss this signal. The approach presented here addresses probabilistic decision making, and the fidelity of decision making in noisy signaling networks. In the particular example of TNF = 8 ng/mL, our approach reveals that there is a 10% chance for a cell not to respond to the signal, based on the measured nuclear NF-κB levels after 30 minutes.

We also note that while our approach is not meant to provide tools to model temporal variations of concentration levels, it allows to analyze and quantify the dynamics of signaling pathways and helps to understand cell decision making processes. In the above example, our approach shows that based on the measured nuclear NF-κB levels after 4 hours of TNF stimulation, the chance for missing the strong signal increases to 29%. This observation agrees with the dynamics of the TNF- NF-κB pathway activity, where due to the negative feedback of A20, the level of nuclear NF-κB decreases after 4 hours, as discussed in the paper.

To further relate the developed approach to the dynamics of signaling, here we have also developed a more sophisticated method to determine cell decision making probabilities, if a cell can make decisions based on the nuclear NF-κB level at the two time points *jointly*, compared to deciding based on 30 minute or 4 hour levels *only*. Our results show that in this example, joint decision based on the two time points has a 10% chance of missing the signal. As discussed in the paper, for this specific pathway, our results suggest that decisions based on joint early/late signaling events versus the early event alone show similar chance for missing the presence of the signal. In other pathways and signaling systems, however, this does not have to be the case, and the presented method can still be used to determine the probability of missing a signal and taking a certain cell fate road, based on multiple observations at different time points.

Overall, the approach complements dynamic modeling by providing quantitative results for assessing the dynamical decision-making performed by a cell in the presence of an external stimulus. In contrast to the more common dynamical modeling analysis, the approach presented here does not explicitly characterize changes in the concentration levels of molecules with time. These approaches are compatible, as a stochastic dynamical model can yield distributions of input-conditioned output levels, expressed in the form of the concentration of a singling molecule of interest. Then our approach can use the simulated concentration level distributions to determine decision thresholds, false alarm and miss probabilities, etc. While it is preferred to use experimental data directly to understand cell decisions, it may be advantageous to use data generated by dynamical models, including those that were developed to describe the TNF-stimulated NF-κB signaling [11]. Furthermore, by perturbing kinetic parameters of a dynamical model, one can investigate the sensitivity of both the concentration level distributions and false alarm and miss probabilities to those parameters. This analysis may reveal that some kinetic parameters can significantly affect cell decisions, while others may play less important roles.

## Conclusion

In summary, the proposed method of the analysis of possible cellular decisions, as applied to the TNF—NF-κB pathway, yields insights that are biologically meaningful and are in agreement with the known pathway functionality. NF-κB is a potent transcription factor regulating expression of numerous genes controlling cell fate decisions, including those regulating proliferation, apoptosis, or transition to the antiviral state. The accuracy of transmitting information between TNF stimulation and NF-κB activation is therefore crucial for proper fate decisions. Based on our analysis we found that the pathway can transmit within 30 minutes the information about the increase of TNF concentration, from a very low level to a high value of 8 ng/mL, with the transmission error of 0.07. Interestingly, when the NF-κB translocation is measured at 4 hours post-stimulation, the transmission error increases to 0.245. This finding reflects the presence of a negative feedback that attenuates the strength of the response at longer times and shifts the TNF-high response histogram to the left (**Fig 1D**). This causes a greater overlap between the two response histograms after 4 hours (**Fig 1D**) and therefore results in a higher decision error probability, compared to that corresponding to the lower overlap between the response histograms after 30 minutes (**Fig 1B**). Consistent with this result, our analysis also indicates a dramatic increase in the decision error in the feedback deficient cells, lacking expression of A20. This implies that cells are not able to compensate for the loss of A20 feedback controlling NF-κB activity. This finding can help account for experimental observations that a loss or mutation of A20 can lead to chronic inflammation and can promote cancer due to the persistent activation of anti-apoptotic genes induced by NF-κB [12].

The decision is expected to become less uncertain with an increasing input dose. Our method can help analyze and quantify this effect. For instance, increasing the TNF dose from 0.2 to 0.51 ng/mL reduces the decision error probability from 0.25 to 0.11 in 30 minute data. The same behavior is observed for 4 hour data.

The method described here can be expanded to describe the performance of more complex and larger signaling networks, including those with multiple ligands or second messengers as network inputs and several transcription factors involved in certain cellular functions as network outputs. By analyzing the concentration levels of these transcription factors using the proposed approach, probabilities of various cell fates in response to the input signals can be computed. We also note that the proposed decision error metrics complement the previously introduced analysis of the information capacity of signaling pathways and networks [2]. The information capacity is a useful metric to study information transmission in signaling pathways, but it does not address how the information transmitted by a signaling network can be converted into cellular decision making. Our results show that the introduced metrics of false alarm, miss and overall error rates can on the other hand be used for modeling decision making errors caused by noise and signaling failures.

Overall, our analysis presents a powerful and widely applicable methodology to evaluate the expected fidelity of cellular decision making that can be used to further evaluate the performance of cellular signaling and communication.

## Materials and methods

### A radar system example: Deciding on the presence of an object generating a constant amplitude signal in background noise [4]

This radar example is presented for illustrative purposes to show how statistical signal processing and decision theory concepts and tools are used in an engineering discipline. It paves the way for understanding the proposed methods and concepts in the context of molecular computational biology and cellular decision making. In radar systems, the system makes a decision based on samples of the received input waveform *x*[*n*], where *n* is the time index. Based on the *N* samples *x*[0],*x*[1],…,*x*[*N*−1], the system should decide between two hypotheses about *x*[*n*]: H_0_ which indicates that only noise is received, i.e., no object is present, and H_1_ which represents that signal plus noise is received, i.e., an object is present. With *w*[*n*] and *A* representing noise and constant amplitude signal, respectively, these two hypotheses can be written as

$$\begin{array}{l} {\text{H}}_{0}:x[n]=w[n],\;n=0,1,\ldots ,N-1,\\ {\text{H}}_{1}:x[n]=A+w[n],\;n=0,1,\ldots ,N-1. \end{array},$$ (1)

To simplify the notation for computing the optimal decision metric, typically it is reasonable to assume both hypotheses have the same probability, i.e., *P*(H_0_) = *P*(H_1_) = 1/2, especially when we do not have a priori information about these probabilities (the case of non-equal probabilities is discussed in the next section). It can be proved [4] that the optimal decision making system which minimizes the decision error probability is the one that compares probabilities of *x* under H_0_ and H_1_. More specifically, let *p*(*x*|H_0_) and *p*(*x*|H_1_) represent conditional probability density functions (PDFs) of *x* under H_0_ and H_1_, respectively. Then the optimal system decides H_1_ if *p*(*x*|H_1_) > *p*(*x*|H_0_), otherwise decides H_0_. This simply means that the optimal decision making system, after observing the input data, picks up the hypothesis which is more probable. This decision strategy is also called the maximum likelihood [4] decision, since it chooses the hypothesis with the highest likelihood.

To compute *p*(*x*|H_0_) and *p*(*x*|H_1_), we need the PDF of noise *w*[*n*]. Upon using a Gaussian noise model with zero mean and variance *σ*^2^ in (1), the univariate conditional PDFs of *x*[*n*] for each *n* under H_0_ and H_1_ can be written as *p*(*x*[*n*]|H_0_) = (2*πσ*^2^)^−1/2^ exp[−(*x*[*n*])^2^/(2*σ*^2^)] and *p*(*x*[*n*]|H_1_) = (2*πσ*^2^)^−1/2^ exp[−(*x*[*n*] − *A*)^2^/(2*σ*^2^)], respectively. These two PDFs are graphed in **S1 Fig** for *A* = 2 and *σ* = 1. When noise samples are independent, joint PDF of *x*[0],*x*[1],…,*x*[*N*−1] becomes the product of individual univariate PDFs. This results in the following expressions for *p*(*x*|H_0_) and *p*(*x*|H_1_)

$$\begin{array}{l} p(x|H_{0})=p(x[0],x[1],\ldots ,x[N-1]|H_{0})={\left(2\pi \sigma^{2}\right)}^{-N/2}\exp[-\sum_{n=0}^{N-1}{\left(x[n]\right)}^{2}/(2\sigma^{2})],\\ p(x|H_{1})=p(x[0],x[1],\ldots ,x[N-1]|H_{1})={\left(2\pi \sigma^{2}\right)}^{-N/2}\exp[-\sum_{n=0}^{N-1}{\left(x[n]-A\right)}^{2}/(2\sigma^{2})]. \end{array}$$ (2)

To compare the above two PDFs, we need to set them equal, to find the optimal decision metric, as well the optimal decision threshold

$$\begin{array}{l} p(x|H_{0})=p(x|H_{1}),\\ \to {\left(2\pi \sigma^{2}\right)}^{-N/2}\exp[-\sum_{n=0}^{N-1}{\left(x[n]\right)}^{2}/(2\sigma^{2})]={\left(2\pi \sigma^{2}\right)}^{-N/2}\exp[-\sum_{n=0}^{N-1}{\left(x[n]-A\right)}^{2}/(2\sigma^{2})],\\ \to \exp[-\sum_{n=0}^{N-1}{\left(x[n]\right)}^{2}/(2\sigma^{2})]=\exp[-\sum_{n=0}^{N-1}{\left(x[n]-A\right)}^{2}/(2\sigma^{2})],\\ \to -\sum_{n=0}^{N-1}{\left(x[n]\right)}^{2}/(2\sigma^{2})=-\sum_{n=0}^{N-1}{\left(x[n]-A\right)}^{2}/(2\sigma^{2}),\\ \to \sum_{n=0}^{N-1}{\left(x[n]-A\right)}^{2}-\sum_{n=0}^{N-1}{\left(x[n]\right)}^{2}=0,\\ \to -2A\sum_{n=0}^{N-1}x[n]+NA^{2}=0,\\ \to N^{-1}\sum_{n=0}^{N-1}x[n]=A/2. \end{array}$$

The above equation indicates that the radar system makes an optimal decision, by comparing the average of *N* observed samples with the optimal threshold *A*/2. It decides H_1_, an object generating a constant signal with amplitude *A* is present, if the average of observed samples is greater than *A*/2

$$\bar{x}=\frac{x[0]+x[1]+\ldots +x[N-1]}{N}>\frac{A}{2},\;{\text{decide}\;\text{H}}_{1}.$$ (3)

Otherwise, the radar decides H_0_, i.e., no object is present and there is only noise.

This optimal radar system still may make mistakes in its decisions due to noise, although the probability of its incorrect decisions is minimized. To calculate the probability of error in making decisions, first we need to calculate probability of deciding H_1_ when H_0_ is true, false alarm probability, and probability of deciding H_0_ when H_1_ is true, i.e., miss probability

$$\begin{array}{c} P_{FA}=P(\text{deciding}\;H_{1}|H_{0}),\\ P_{M}=P(\text{deciding}\;H_{0}|H_{1}). \end{array}$$

To compute the above probabilities, we need to determine the PDF of the decision variable $\bar{x}=N^{-1}\sum_{n=0}^{N-1}x[n]$ introduced earlier, under the two hypotheses. As discussed previously and under H_0_, *x*[0],*x*[1],…,*x*[*N*−1] are noise samples, independent and Gaussian with zero mean and variance *σ*^2^. Using properties of Gaussian random variables, it can be shown that $\bar{x}$ here is Gaussian with zero mean and variance *σ*^2^/*N*

$$p(\bar{x}|H_{0})={\left(2\pi \sigma^{2}/N\right)}^{-1/2}\exp[-{\bar{x}}^{2}/\left(2\sigma^{2}/N\right)].$$

Under H_1_, on the other hand, *x*[0],*x*[1],…,*x*[*N*−1] are signal plus noise samples, independent and Gaussian with mean *A* and variance *σ*^2^. Using properties of the sum of Gaussian random variables, it can be shown that now $\bar{x}$ is Gaussian with mean *A* and variance *σ*^2^/*N*

$$p(\bar{x}|H_{1})={\left(2\pi \sigma^{2}/N\right)}^{-1/2}\exp[-{\left(\bar{x}-A\right)}^{2}/\left(2\sigma^{2}/N\right)].$$

To compute *P*_*FA*_, we note that false alarm occurs when H_0_ is true, but according to Eq (3) we have $\bar{x}>{\bar{x}}_{th}$, where ${\bar{x}}_{th}=A/2$. This results in

$$P_{FA}=P(\bar{x}>{\bar{x}}_{th}|H_{0})=\int_{{\bar{x}}_{th}}^{\infty }p(\bar{x}|H_{0})d\bar{x}.$$

Integrating the expression for $p(\bar{x}|H_{0})$, derived earlier, provides us with the following formula for the false alarm probability

$$P_{FA}=Q\left(\frac{\sqrt{N}{\bar{x}}_{th}}{\sigma }\right),$$

where *Q* is a commonly-used Gaussian probability function

$$Q(\eta )={\left(2\pi \right)}^{-1/2}\int_{\eta }^{\infty }\exp(-u^{2}/2)du.$$

To compute *P*_*M*_, we similarly note that miss occurs when H_1_ is true, but we have $\bar{x}<{\bar{x}}_{th}$. This results in

$$P_{M}=P(\bar{x}<{\bar{x}}_{th}|H_{1})=\int_{-\infty }^{{\bar{x}}_{th}}p(\bar{x}|H_{1})d\bar{x}.$$

Integration of the expression for $p(\bar{x}|H_{1})$, derived earlier, gives the following formula for the miss probability in terms of the *Q* function

$$P_{M}=Q\left(\frac{\sqrt{N}(A-{\bar{x}}_{th})}{\sigma }\right).$$

The overall probability of error in making decisions by the radar system is a mixture of false alarm and miss probabilities

$$P_{e}=P(H_{0})P(\text{deciding}\;H_{1}|H_{0})+P(H_{1})P(\text{deciding}\;H_{0}|H_{1})=P(H_{0})P_{FA}+P(H_{1})P_{M}.$$

By substituting *P*(H_0_) = *P*(H_1_) = 1/2, and *P*_*FA*_ and *P*_*M*_ formulas, finally the probability of error can be written as

$$P_{e}=\frac{1}{2}Q\left(\frac{\sqrt{N}{\bar{x}}_{th}}{\sigma }\right)+\frac{1}{2}Q\left(\frac{\sqrt{N}(A-{\bar{x}}_{th})}{\sigma }\right).$$

The above formula holds true for the optimal threshold ${\bar{x}}_{th}=A/2$, as well as other choices for ${\bar{x}}_{th}$. To understand the importance of the decision threshold and how it affects *P*_*e*_, the above formula is graphed in **S2 Fig** versus ${\bar{x}}_{th}$, for *A* = 2, *σ* = 1 and *N* = 4. We observe that the probability of error is minimal when ${\bar{x}}_{th}$ is the optimal threshold of *A*/2 = 1, and departure of the decision threshold from the optimal value increases *P*_*e*_.

With the choice of the optimal threshold, ${\bar{x}}_{th}=A/2$, the above *P*_*e*_ formula simplifies to

$$P_{e}=Q\left(\frac{\sqrt{N}A}{2\sigma }\right).$$

This formula is graphed in **S3 Fig** versus the signal-to-noise ratio *A*/*σ*, for *N* = 4. We observe that the probability of error in making decisions decreases as signal-to-noise ratio increases, as expected.

### Optimal maximum likelihood decision, false alarm, miss and overall decision error probabilities in a cell

Making a decision on whether TNF level at the signaling system input is high or low is a binary hypothesis testing problem. The two hypotheses are H_1_: TNF is high, and H_0_: TNF is low. Due to the signal transduction noise or signaling malfunctions in a cell, it can respond differently to the same input, which may result in incorrect (unexpected) cell decisions and responses. Cell can make two types of incorrect decisions: deciding that TNF is high at the system input whereas in fact it is low (deciding H_1_ when H_0_ is true), and missing TNF’s high level when it is actually high (deciding H_0_ when H_1_ is true). These two incorrect decisions can be called false alarm and miss events, respectively.

Let *x* be the measured quantity based on which the decision is going to be made. With *p*(*x*|H_0_) and *p*(*x*|H_1_) as the conditional probability density functions (PDFs) of *x* under H_0_ and H_1_, respectively, false alarm and miss probabilities can be written as [4]

$$P_{FA}=\int_{x\in false\;alarm\;region}^{}p(x|H_{0})dx,$$ (4)

$$P_{M}=\int_{x\in miss\;region}^{}p(x|H_{1})dx,$$ (5)

where false alarm and miss regions will be specified later. The overall probability of error *P*_*e*_ for making a decision is given by

$$P_{e}=P(H_{0})P_{FA}+P(H_{1})P_{M},$$ (6)

where *P*(H_0_) and *P*(H_1_) are probabilities of H_0_ and H_1_, respectively. It can be shown [4] the optimal decision making system that minimizes the decision error probability *P*_*e*_ is the one that compares the conditional likelihood ratio *L*(*x*) = *p*(*x*|H_1_)/*p*(*x*|H_0_) with the ratio *γ* = *P*(H_0_)/*P*(H_1_). The optimal system decides H_1_ if *L*(*x*) > *γ*. When H_0_ and H_1_ are equi-probable, *P*(H_0_) = *P*(H_1_) = 1/2, the optimal decision decides H_1_ if *L*(*x*) > 1, which means comparing the two conditional PDFs

$$p(x|H_{1})>p(x|H_{0}),\;\mathrm{decide}\;{\text{H}}_{1}.$$ (7)

This decision rule is called the maximum likelihood [4] decision, since it chooses the hypothesis with the highest likelihood. The choice of *P*(H_0_) = *P*(H_1_) = 1/2 represents the case where a priori knowledge on the probabilities of H_0_ and H_1_ is not available. This is considered just to demonstrate the proposed method. When *P*(H_0_) and *P*(H_1_) are known, the maximum likelihood decision rule simply changes to *P*(H_1_)*p*(*x*|H_1_) > *P*(H_0_)*p*(*x*|H_0_), to decide H_1_.

### Computing false alarm and miss decision probabilities in the TNF—NF-κB system based on early or late event data

To evaluate the performance of the maximum likelihood decision, we need to compute its false alarm and miss probabilities in the signaling system, which according to Eqs (4) and (5) can be written as

$$P_{FA}=\int_{\left\{x:p(x|H_{1})>p(x|H_{0})\right\}}p(x|H_{0})dx,$$ (8)

$$P_{M}=\int_{\left\{x:p(x|H_{0})>p(x|H_{1})\right\}}p(x|H_{1})dx.$$ (9)

In these formulas the PDFs *p*(*x*|H_0_) and *p*(*x*|H_1_) represent the response probabilities of NF-κB nuclear translocation when TNF level is low and high, respectively. Similarly to Cheong et al. [2] we consider the Gaussian PDF *p*(*x*) = (2*πσ*^2^)^−1/2^ exp[−(*x*−*μ*)^2^/(2*σ*^2^)] for the nuclear NF-κB level (**Fig 1C**, **Fig 1E**), where *μ* and *σ*^2^ are the mean and variance, respectively. We symbolically represent this by *x* ∼ N(*μ*,*σ*^2^), where N stands for the Normal or Gaussian PDF. To determine *P*_*FA*_ and *P*_*M*_, false alarm and miss integration regions in Eqs (8) and (9) should be specified, by solving the equation *p*(*x*|H_0_) = *p*(*x*|H_1_). Since these two PDFs are $N(\mu_{0},\sigma_{0}^{2})$ and $N(\mu_{1},\sigma_{1}^{2})$, respectively, equating them provides the following equation

$$\begin{array}{l} {\left(2\pi \sigma_{0}^{2}\right)}^{-1/2}\exp[-{\left(x-\mu_{0}\right)}^{2}/\left(2\sigma_{0}^{2}\right)]={\left(2\pi \sigma_{1}^{2}\right)}^{-1/2}\exp[-{\left(x-\mu_{1}\right)}^{2}/\left(2\sigma_{1}^{2}\right)],\\ \to \frac{\exp[-{\left(x-\mu_{0}\right)}^{2}/\left(2\sigma_{0}^{2}\right)]}{\exp[-{\left(x-\mu_{1}\right)}^{2}/\left(2\sigma_{1}^{2}\right)]}=\frac{{\left(2\pi \sigma_{1}^{2}\right)}^{-1/2}}{{\left(2\pi \sigma_{0}^{2}\right)}^{-1/2}},\\ \to \exp[-{\left(x-\mu_{0}\right)}^{2}/\left(2\sigma_{0}^{2}\right)+{\left(x-\mu_{1}\right)}^{2}/\left(2\sigma_{1}^{2}\right)]=\sigma_{0}/\sigma_{1}. \end{array}$$

By taking the natural logarithm of both sides of the above last equation we obtain

$$\begin{array}{l} -{\left(x-\mu_{0}\right)}^{2}/\left(2\sigma_{0}^{2}\right)+{\left(x-\mu_{1}\right)}^{2}/\left(2\sigma_{1}^{2}\right)=\ln(\sigma_{0}/\sigma_{1}),\\ \to \frac{\sigma_{0}^{2}{\left(x-\mu_{1}\right)}^{2}-\sigma_{1}^{2}{\left(x-\mu_{0}\right)}^{2}}{2\sigma_{0}^{2}\sigma_{1}^{2}}=\ln(\sigma_{0}/\sigma_{1}),\\ \to \sigma_{0}^{2}{\left(x-\mu_{1}\right)}^{2}-\sigma_{1}^{2}{\left(x-\mu_{0}\right)}^{2}=2\sigma_{0}^{2}\sigma_{1}^{2}\ln(\sigma_{0}/\sigma_{1}), \end{array}$$

which can be re-written in the form of the following quadratic equation

$$(\sigma_{0}^{2}-\sigma_{1}^{2})x^{2}+2(\sigma_{1}^{2}\mu_{0}-\sigma_{0}^{2}\mu_{1})x+\sigma_{0}^{2}\mu_{1}^{2}-\sigma_{1}^{2}\mu_{0}^{2}-2\sigma_{0}^{2}\sigma_{1}^{2}\ln(\sigma_{0}/\sigma_{1})=0,$$ (10)

where ln(.) is the natural logarithm. As mentioned previously, Eq (10) is derived assuming *P*(H_0_) = *P*(H_1_) = 1/2, i.e., equal probabilities for having low and high TNF levels, and considering a Gaussian model for the nuclear NF-κB level. For other prior probabilities and distribution models, the threshold can be similarly obtained, by solving the equation *P*(H_0_)*p*(*x*|H_0_) = *P*(H_1_)*p*(*x*|H_1_) for *x*. The solution to the quadratic Eq (10) gives *NFκB*_*th*_, the threshold value of NF-κB, such that *p*(*NFκB*_*th*_|H_0_) = *p*(*NFκB*_*th*_|H_1_) (**Fig 1C**, **Fig 1E**). By computing the integrals in Eqs (8) and (9), as shown below, we obtain the following results for false alarm and miss probabilities

$$P_{FA}=\int_{NF\kappa B_{th}}^{\infty }p(x|H_{0})dx=Q\left(\frac{NF\kappa B_{th}-\mu_{0}}{\sigma_{0}}\right),$$ (11)

$$P_{M}=\int_{-\infty }^{NF\kappa B_{th}}p(x|H_{1})dx=Q\left(\frac{\mu_{1}-NF\kappa B_{th}}{\sigma_{1}}\right),$$ (12)

where *Q* function is defined as

$$Q(\eta )={\left(2\pi \right)}^{-1/2}\int_{\eta }^{\infty }\exp(-u^{2}/2)du.$$ (13)

To measure *P*_*FA*_ and *P*_*M*_, we used single cell data collected from hundreds of cells [2], to estimate $\left(\mu_{0},\sigma_{0}^{2})\;\mathrm{and}\;(\mu_{1},\sigma_{1}^{2}\right)$ of nuclear NF-κB readouts after 30 minutes (early events), for low and high TNF levels, 0.0021 ng/mL and 8 ng/mL, respectively. Then using Eq (10) we estimated the decision threshold *NFκB*_*th*_ (**Fig 1C**) which upon substituting into Eqs (11) and (12) resulted in the false alarm and miss probabilities *P*_*FA*_ = 0.04 and *P*_*M*_ = 0.1, respectively. Repeating the same steps for nuclear NF-κB readouts after 4 hours (late events) resulted in a decision threshold *NFκB*_*th*_ (**Fig 1E**) which after substitution into Eqs (11) and (12) provided *P*_*FA*_ = 0.2 and *P*_*M*_ = 0.29, respectively.

Overall, in this study we have made the following assumptions, which can be relaxed, as explained below: Probabilities of having different input signals, i.e., low and high TNF levels herein, are equal; and, concentration level of interest, which is nuclear NF-κB level in our work, has a Gaussian distribution.

The first assumption is for cases where a priori knowledge on these probabilities is not available. The developed method, however, is not limited to this assumption and can incorporate non-equal prior probabilities, if they become available. If a priori probabilities are not equal, the threshold can be determined by comparing *P*(H_1_)*p*(*x*|H_1_) and *P*(H_0_)*p*(*x*|H_0_), rather than *p*(*x*|H_1_) and *p*(*x*|H_0_). The overall probability of error in making decisions also changes from *P*_*e*_ = (1/2)*P*_*FA*_ + (1/2)*P*_*M*_ to *P*_*e*_ = *P*(H_0_)*P*_*FA*_ + *P*(H_1_)*P*_*M*_.

The second assumption is made following the study of Cheong et al. [2], which has considered a Gaussian model for the nuclear NF-κB level. This model reasonably represents the data. For other data sets and other distribution models, one can still use the developed approach, using modified mathematical formulas for the decision threshold, false alarm and miss probabilities, obtained by integrating the probability distribution of interest. More specifically, we have obtained the decision threshold by solving the equation *p*(*x*|H_0_) = *p*(*x*|H_1_) for *x*. When they are both Gaussian, the equation simplifies to the quadratic Eq (10). For a non-Gaussian distribution, we will obtain another equation to compute the threshold, still by solving the equation *p*(*x*|H_0_) = *p*(*x*|H_1_) for *x*. Additionally, integration of a non-Gaussian distribution to obtain false alarm and miss probabilities using Eqs (11) and (12) will give us results that will be different from the *Q* function. If the data is not easily characterized by a well-known distribution, one can model the data using various probability density function estimators. Alternatively, one can estimate threshold value and false alarm and miss probabilities directly from empirical histograms.

The derived formulas for false alarm and miss error probabilities in the NF-κB pathway, Eqs (11) and (12), show some biological factors such as mean expression levels of NF-κB and its noise-induced variances that affect decision makings. For example, since the *Q* function is inversely related to its argument, we note that as variances increase, the overall decision error probability can increase. This is biologically relevant, as larger variances broaden NF-κB response curves, which in turn cause more overlap between the response curves, therefore resulting in a higher decision error probability.

To understand the effect of various components of the pathway on decision making, one can knockout or knockdown these components and calculate decision error probabilities in the modified system, as we did in A20^-/-^ cells.

### Optimal maximum likelihood decision in the TNF—NF-κB system based on both early and late event data, and computing its false alarm and miss decision probabilities

Maximum likelihood decision based on the data at two time points needs the joint PDF of *x* and *y*, which represent the nuclear NF-κB level after 30 minutes and 4 hours, respectively. The joint Gaussian PDF is given by [13]

$$p(x,y)=\frac{1}{2\;\pi \sigma_{x}\sigma_{y}\sqrt{1-\rho^{2}}}\;\exp\left(-\frac{1}{2(1-\rho^{2})}\left[\frac{{\left(x-\mu_{x}\right)}^{2}}{\sigma_{x}^{2}}-\frac{2\rho (x-\mu_{x})(y-\mu_{y})}{\sigma_{x}\sigma_{y}}+\frac{{\left(y-\mu_{y}\right)}^{2}}{\sigma_{y}^{2}}\right]\right),$$ (14)

where *ρ* is the correlation coefficient between *x* and *y*, whereas $\left(\mu_{x},\sigma_{x}^{2}\right)$ and $\left(\mu_{y},\sigma_{y}^{2}\right)$ are the mean and variance of *x* and *y*, respectively. Upon defining the following mean vector **μ** and covariance matrix **Σ** for *x* and *y*

$$\mu =\left[\begin{array}{c} \mu_{x}\\ \mu_{y} \end{array}\right],\;\Sigma =\left[\begin{array}{cc} \sigma_{x}^{2} & \rho \sigma_{x}\sigma_{y}\\ \rho \sigma_{x}\sigma_{y} & \sigma_{y}^{2} \end{array}\right],$$ (15)

we succinctly represent the joint Normal or Gaussian PDF in Eq (14) for (*x*,*y*) by the notation (*x*,*y*) ∼ N(**μ**,**Σ**). To determine *ρ*, we used an experimentally-verified simulator [3] whose accuracy is verified by single cell data [3]. To evaluate the performance of the maximum likelihood decision based on early and late event data, we need to compute its false alarm and miss probabilities in the signaling system, by extending Eqs (8) and (9) to two variables

$$P_{FA}=\iint_{\left\{x,y:p(x,y|H_{1})>p(x,y|H_{0})\right\}}p(x,y|H_{0})dxdy,$$ (16)

$$P_{M}=\iint_{\left\{x,y:p(x,y|H_{0})>p(x,y|H_{1})\right\}}p(x,y|H_{1})dxdy,$$ (17)

where the bivariate PDFs *p*(*x*,*y*|H_0_) = N(**μ**_0_,**Σ**_0_) and *p*(*x*,*y*|H_1_) = N(**μ**_1_,**Σ**_1_) represent the joint early/late response probabilities of NF-κB nuclear translocation when TNF level is low and high, respectively (**Fig 1F**). To find the integration regions in Eqs (16) and (17), we need to solve the equation *p*(*x*,*y*|H_0_) = *p*(*x*,*y*|H_1_). The solution is a threshold curve in the (*x*,*y*) plane. Performing the double integrations in Eqs (16) and (17), however, is not straightforward either analytically or numerically. Therefore, we resorted to Monte Carlo integration which resulted in *P*_*FA*_ = 0.03 and *P*_*M*_ = 0.1.

### Computing false alarm and miss decision probabilities in the TNF—NF-κB system for A20^-/-^ cells

Similarly to wild-type cells, we considered Gaussian PDF for the nuclear NF-κB level in A20^-/-^ cells (**Fig 2A**, **Fig 2C**). Upon using the same steps and equations and thresholds as wild-type cells, we computed *P*_*FA*_ and *P*_*M*_ in A20^-/-^ cells (**Fig 2B**).

## Supporting information

S1 Dataset All the data analyzed in the paper. (MAT)

S1 Fig (PDF)

S2 Fig (PDF)

S3 Fig (PDF)
