# Supplementary material for: Computation and measurement of cell decision making errors using single cell data
Source: PLoS Comput Biol. 2017 Apr 5;13(4):e1005436. doi: 10.1371/journal.pcbi.1005436 (PMC5397092; doi:10.1371/journal.pcbi.1005436)
Supplement: S1 Fig — (PDF) [file pcbi.1005436.s002.pdf]

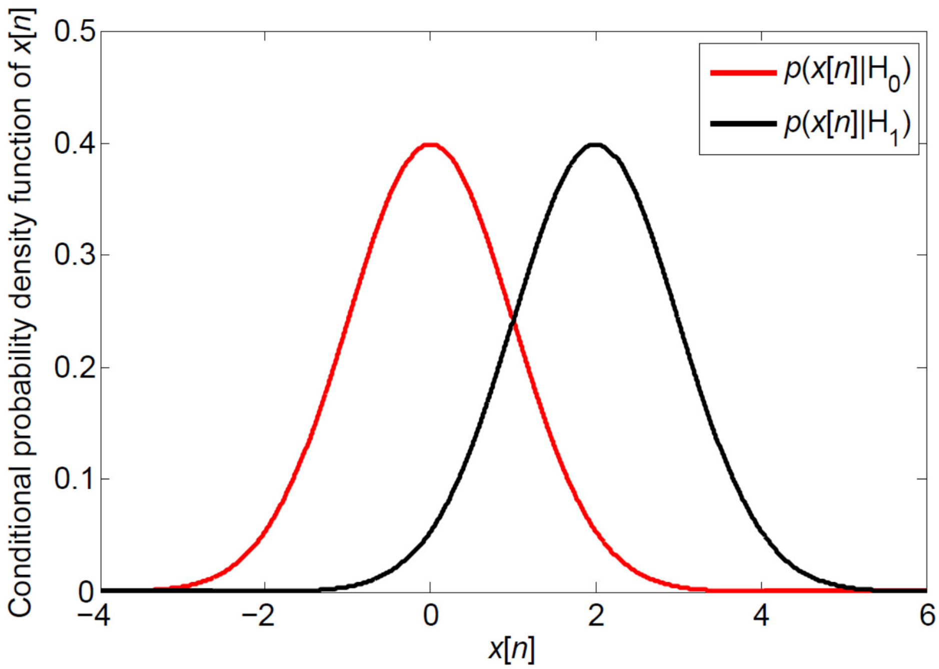


Figure S1.

Examples of probability density functions of the waveform $x[n]$ that a radar system may receive. The $\mathrm{H}_0$ graph represents the case where only Gaussian noise is received, i.e., no object is present, and the $\mathrm{H}_1$ graph stands for the case where a constant signal with amplitude $A = 2$ plus Gaussian noise is received, i.e., an object is present.
