# Supplementary material for: Computation and measurement of cell decision making errors using single cell data
Source: PLoS Comput Biol. 2017 Apr 5;13(4):e1005436. doi: 10.1371/journal.pcbi.1005436 (PMC5397092; doi:10.1371/journal.pcbi.1005436)
Supplement: S2 Fig — (PDF) [file pcbi.1005436.s003.pdf]

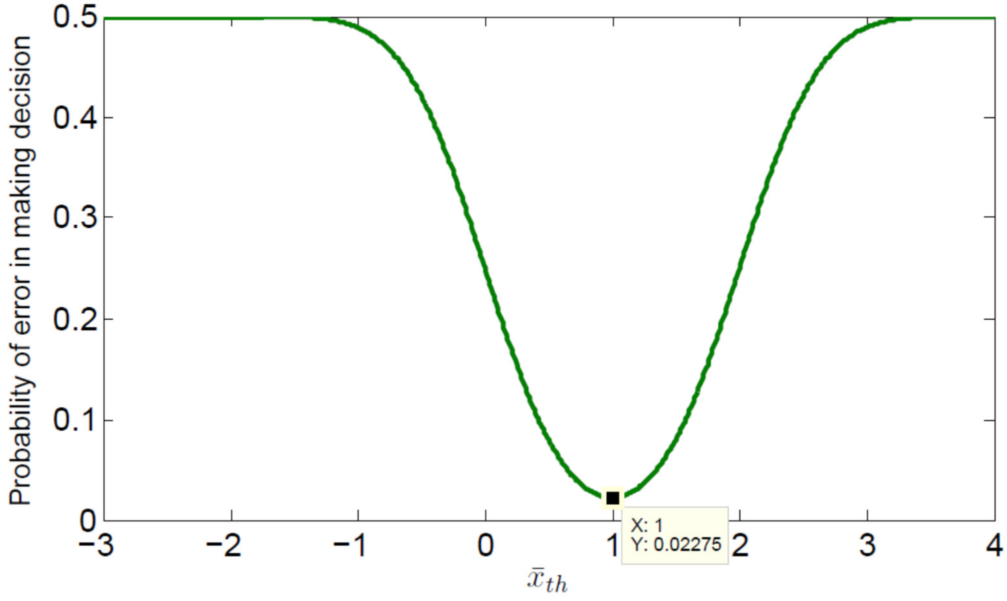

Figure S2.

Probability of error for deciding on the presence of a constant amplitude signal A in noise by a radar system, where the decision threshold $\bar{x}_{th}$ is not necessarily the optimal threshold $A/2$. To make the figure, $A = 2$ is considered.
