# Supplementary material for: Computation and measurement of cell decision making errors using single cell data
Source: PLoS Comput Biol. 2017 Apr 5;13(4):e1005436. doi: 10.1371/journal.pcbi.1005436 (PMC5397092; doi:10.1371/journal.pcbi.1005436)
Supplement: S3 Fig — (PDF) [file pcbi.1005436.s004.pdf]

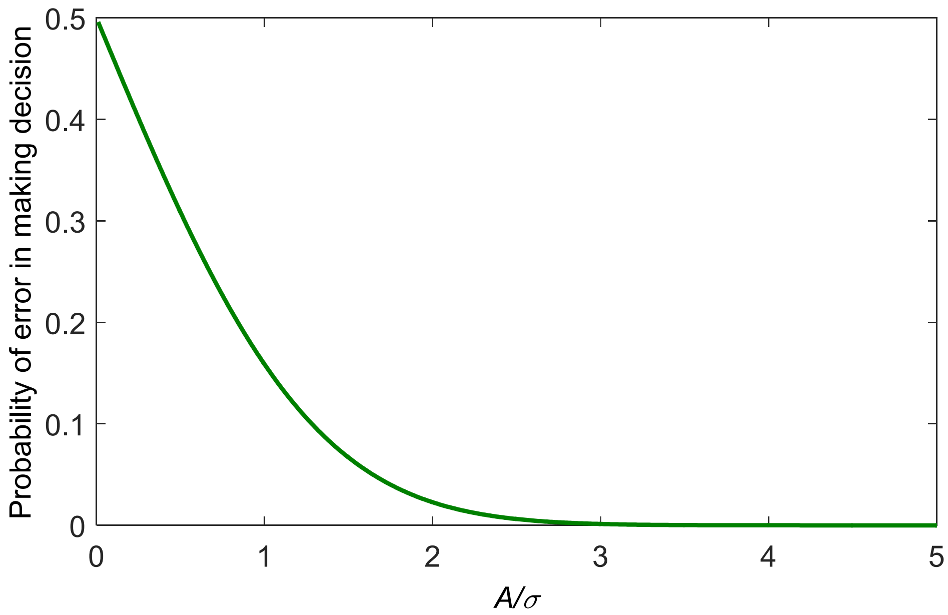

Figure S3.

Radar system probability of error for deciding on the presence of a constant amplitude signal A in noise with power σ^2, versus the signal-to-noise ratio A/σ.
